# Supplementary material for: Two angles of overqualification-the deviant behavior and creative performance: The role of career and survival job
Source: PLoS One. 2020 Jan 2;15(1):e0226677. doi: 10.1371/journal.pone.0226677 (PMC6940141; doi:10.1371/journal.pone.0226677)
Supplement: S1 Questionnaire — (DOCX) [file pone.0226677.s001.docx]

**Questionnaire**

**(Overqualified Teachers)**

**Dear respondent,**

I am a PhD student at the Department of Business Administration, *Sarhad University of Science and IT Peshawar, Pakistan*. Dr. Wali Rahman of the same university is co-researcher with me. We are conducting a study on overqualified employees with the aim to know as to what extent their overqualification affects the stakes of all stakeholders in an organization. For this purpose I need your cooperation and participation will be of tremendous help to this research.

In the subject area we want to know your personal opinion. Participation is voluntary. Let me assure you that this is a purely academic pursuit and is research oriented. Your responses in this questionnaire will be held in the strictest confidence. All data collected from this questionnaire will be analyzed and reported as anonymously.

Please answer all questions candidly and return it to the researcher on the address given below. If you have any concern or question(s), please feel free in your expression.

Your time sparing and cooperation is, once again, whole heartedly appreciated!

Yours sincerely,

Nasib Dar,

Ph.D. Scholar (Email: [nas685@yahoo.com](mailto:nas685@yahoo.com))

**Demographic Information (Tick the relevant box)**

| **Gender:** | Male | Female | Other |
| --- | --- | --- | --- |
| **Marital status:** | Single   | Married   | Divorce   |
| **Age:** | < 30 years   | 30 – 50 years   | 50 + years   |
| **Education:** | Master   | MS/M Phil   | PhD   |
| **Experience** | < 5 years   | 5 – 10 years   | 10 +   |

**Career Stakes**

***How is your current job related to your long-term career goals? (Tick only one)***

1. Career job
2. Survival job

**Perceived overqualification** (Hints: Strongly Disagree (SD) = 1; Disagree (D) = 2; Neither Agree nor Disagree (N) = 3; Agree (A) = 4; Strongly Agree (SA) = 5

| **#** | **Description** | **SD** | **D** | **N** | **A** | **SA** |
| --- | --- | --- | --- | --- | --- | --- |
| 1. | My job requires less education than I have. | 1 | 2 | 3 | 4 | 5 |
| 2. | The work experience that I have is not necessary to be successful on this job. | 1 | 2 | 3 | 4 | 5 |
| 3. | I have job skills that are not required for this job. | 1 | 2 | 3 | 4 | 5 |
| 4. | Someone with less education than me could perform well on my job. | 1 | 2 | 3 | 4 | 5 |
| 5. | My previous training is not being fully utilized on this job. | 1 | 2 | 3 | 4 | 5 |
| 6. | My education level is above the education level required by my job. | 1 | 2 | 3 | 4 | 5 |
| 7. | Someone with less work experience than me could do my job just well. | 1 | 2 | 3 | 4 | 5 |
| 8. | I have a lot of knowledge that I do not need in order to do my job. | 1 | 2 | 3 | 4 | 5 |
| 9. | I have more abilities than I need in order to do my job. | 1 | 2 | 3 | 4 | 5 |

**Deviant workplace behavior**

| **#** | **Description** | **SD** | **D** | **N** | **A** | **SA** |
| --- | --- | --- | --- | --- | --- | --- |
| 1. | I have taken official property from school without permission. | 1 | 2 | 3 | 4 | 5 |
| 2. | I have spent too much time fantasizing or daydreaming instead of working. | 1 | 2 | 3 | 4 | 5 |
| 3. | I have falsified a receipt to get reimbursed for more money than I spent on school different expenses. | 1 | 2 | 3 | 4 | 5 |
| 4. | I have taken an additional or longer break than is acceptable at my school/department. | 1 | 2 | 3 | 4 | 5 |
| 5. | I come late to school without permission. | 1 | 2 | 3 | 4 | 5 |
| 6. | I have neglected to follow my head's instructions. | 1 | 2 | 3 | 4 | 5 |
| 7. | I intentionally worked slower than I could have worked. | 1 | 2 | 3 | 4 | 5 |
| 8. | I discussed confidential institution/school information with an unauthorized person. | 1 | 2 | 3 | 4 | 5 |
| 9. | I made fun of someone at school. | 1 | 2 | 3 | 4 | 5 |
| 10. | I made an ethnic, religious, or racial remark at school. | 1 | 2 | 3 | 4 | 5 |
| 11. | I acted rudely toward someone at school. | 1 | 2 | 3 | 4 | 5 |
| 12. | I publicly embarrassed someone at school. | 1 | 2 | 3 | 4 | 5 |
