# Supplementary material for: Two angles of overqualification-the deviant behavior and creative performance: The role of career and survival job
Source: PLoS One. 2020 Jan 2;15(1):e0226677. doi: 10.1371/journal.pone.0226677 (PMC6940141; doi:10.1371/journal.pone.0226677)
Supplement: S2 Questionnaire — (DOCX) [file pone.0226677.s002.docx]

**Questionnaire**

**(Students)**

**Dear respondent,**

I am a PhD student at the Department of Business Administration, *Sarhad University of Science and IT Peshawar, Pakistan*. Dr. Wali Rahman of the same university is co-researcher with me. We are conducting a study on overqualified employees with the aim to know as to what extent their overqualification affects the stakes of all stakeholders in an organization. For this purpose I need your cooperation and participation will be of tremendous help to this research.

In the subject area we want to know your personal opinion. Participation is voluntary. Let me assure you that this is a purely academic pursuit and is research oriented. Your responses in this questionnaire will be held in the strictest confidence. All data collected from this questionnaire will be analyzed and reported as anonymously.

Please answer all questions candidly and return it to the researcher on the address given below. If you have any concern or question(s), please feel free in your expression.

Your time sparing and cooperation is, once again, whole heartedly appreciated!

Yours sincerely,

Nasib Dar,

Ph.D. Scholar (Email: [nas685@yahoo.com](mailto:nas685@yahoo.com))

**(Structured Interview)**

**Demographic Information (Tick the relevant box)**

| **Gender:** | Male | Female | Other |
| --- | --- | --- | --- |

***Questions used in structured interview from target students***

Hints: Strongly Disagree (SD) = 1; Disagree (D) = 2; Neither Agree nor Disagree (N) = 3;

Agree (A) = 4; Strongly Agree (SA) = 5

It was observed that to what extent a student agree/disagree with each statement/question and thus recorded according to above hints.

**Creative performance**

1. **How creative is this teacher's work?**

*Creativity refers to the extent to which the employee develops ideas, methods, or products that are originaland useful to the organization.*

1. **How original and practical is this teacher's work?**

*Original and practical work refers to developing ideas, methods, or products that are both totally unique and especially useful to the organization.*

1. **How adaptive and practical is this teacher's work?**

*Adaptive and practical work refers to using existing information or materials to develop ideas, methods, or products that are useful to the organization.*

1. **Does this teacher search out new processes, techniques, and/or new ideas for his/her classes?**
2. **Does this teacher, mostly, come with creative ideas?**
