# Supplementary material for: Two angles of overqualification-the deviant behavior and creative performance: The role of career and survival job
Source: PLoS One. 2020 Jan 2;15(1):e0226677. doi: 10.1371/journal.pone.0226677 (PMC6940141; doi:10.1371/journal.pone.0226677)
Supplement: S1 Data — (ZIP) [file pone.0226677.s003.zip › Fig 1.docx]

**Supporting information**

**S1 Fig. Hypothesized model**

Perceived over-qualification

Perception of job (career or survival job)

Deviant

behaviors

Creative

performance
