# Supplementary material for: Two angles of overqualification-the deviant behavior and creative performance: The role of career and survival job
Source: PLoS One. 2020 Jan 2;15(1):e0226677. doi: 10.1371/journal.pone.0226677 (PMC6940141; doi:10.1371/journal.pone.0226677)
Supplement: S1 Data — (ZIP) [file pone.0226677.s003.zip › Fig 2.docx]

**Supporting information**

**S2 Fig. The interaction effect of perception of job type (career or survival job) and overqualification on creative performance.**

Low Overqualification

Creative

Performance

■ Career job

**₋ ₋ ■ ₋ ₋** Survival job

High Overqualification
