# Supplementary material for: Two angles of overqualification-the deviant behavior and creative performance: The role of career and survival job
Source: PLoS One. 2020 Jan 2;15(1):e0226677. doi: 10.1371/journal.pone.0226677 (PMC6940141; doi:10.1371/journal.pone.0226677)
Supplement: Attachment — Submitted filename: Response to Reviewers.docx [file pone.0226677.s005.docx]

**Compliance Report**

PONE-D-19-21532
**Deviant behavior and creative performance—the two outcomes of overqualification: The role of career and survival job.**

| **#** | **PLOS Questions** | **Reviewer’s Response** | **Response of the Authors** |
| --- | --- | --- | --- |
| 1. | Is the manuscript technically sound, and do the data support the conclusions? | Reviewer 1= Partly | The authors have gone through the whole paper again and again to address this aspect of the paper. In this effort, additional text has been added. |
|  |  | Reviewer 2= Partly |  |
| 2 | Has the statistical analysis been performed appropriately and rigorously? | Reviewer 1= Yes | NA |
|  |  | Reviewer 2= Yes | NA |
| 3. | Have the authors made all data underlying the findings in their manuscript fully available? | Reviewer 1= No | Data file can be provided when asked for. Authors do not feel any reservations on sharing it with the reviewers and with editors. |
|  |  | Reviewer 2= No |  |
| 4. | Is the manuscript presented in an intelligible fashion and written in standard English? | Reviewer 1= No | Major portion of the manuscript has been revamped to make it in line with the standard language. Though English is not our first language, we believe that our language is functional if it is not of high standard. We say it with some surety because our whole education is in English and have writing in English since long. |
|  |  | Reviewer 2= No |  |

**Comments to the Author (Reviewer # 1)**

| **#** | **Comments/Observations of the Reviewer** | **Response of the Authors** |
| --- | --- | --- |
| 1. | The manuscript (MS) ‘Deviant behavior and creative performance—the two outcomes of overqualification: The role of career and survival job’ presents a study in a non-WEIRD sample in which hypothesis are tested relating consequences of the overqualification with two criterion variables: deviant behavior and creative performance. The MS subject is relevant mainly because the research was done in substituted culture concerning this and other subjects typically researched in organizational psychology. The MS has contributions to the literature, but in its present shape it lacks some main issues, which lead me to recommend a revision. | NA |
| 2. | The MS has some central conceptual flaws, such as a clear and explicit definition of deviant behavior and creative performance. The author argues that the use of students as source of information is a contribution to the field of research, but there is no clarity of which measure was applied to students and how it was operationalized. For instance, the paragraph between lines 252-258 that should describe such measure is not clear and produces misunderstandings in the reader when there is mention of supervisor ratings. Which supervisors? | To explain the concept of deviant behaviour, a full paragraph (lines 120 to 134) has been added. Efforts have been undertaken to operationalize this concept in the light of overqualification. |
| 3. | It is also important to mention that the description of H3 and H4 are inaccurate, considering both hypotheses are impossible to falsify, considering it describe all possible relationships that variables can have. Concerning authors used measures built in different cultures is essential that they providence evidences of cultural adaptation of such measures, considering many different problems that such emerge to this kind of use, such as imposed-ethics. | Description of the H3 and H4 has been addressed to make it more logically connected and easy to understand. |
| 4. | A minor issue: the MS deserve a careful revision of spelling errors, because there are several throughout the MS. | The whole MS has been proofread and a number of concord and other semantic and syntactic issues have been addressed as per understanding of the authors. |

**Comments to the Author (Reviewer # 2)**

| # | **Comments/Observations of the Reviewer** | **Response of the Authors** |
| --- | --- | --- |
| 1. | The paper deals with a very interesting and quite original topic that would be certainly of interest for the readership of the journal: the relationship between overqualification perception and creative performance and deviant behaviors in the workplace. Though an overall positive appreciation I think that the MS has some weaknesses that need to be addressed. First of all, the theoretical section needs to be enriched by a more extensive description of the constructs considered. The authors have articulated this introductory section into a literature review that carefully considered the single relationships between overqualification and creative performance and overqualification and deviant behaviors. However, there is neither clear definition of deviant behavior nor of creative performance. Also career or survival job is not clearly defined. Further I am not fully convinced about H3 and H4 that sound ambiguous since they consider both positive and negative relationships at the same time. Finally, participants to the study have been both students and teachers. But actually I cannot find reference to how and why students been involved in a study related to overqualification. The conclusion section is too short. I will suggest making a single section discussion and conclusion and then practical implications and future research. Language should also be improved. There are a number of misspelled words. I suggest a native speaker check. | The authors have tried their level best to address the weakness of the paper as pointed out by the reviewers.  Ambiguities in terms of expression in the theoretical section have been addressed.  To define the concepts of creative performance, overqualification, and deviant behavior, a full paragraph (lines 120-134) has been added.  Similarly, H3 and H4 are aggregate hypotheses. They have sub-hypotheses that H3a and H3b, H4a and H4b respectively. In the figure they are in the same model but actually, they are separately tested and results are discussed.  Students have been involved only to have their responses on creativity of the teachers. As they are the direct beneficiaries of their respective teacher creativity, therefore, we took their perception into consideration. In the past, this aspect has generally been assessed through their respective supervisor, we changed supervisors and took students to have more realistic picture.  So far conciseness of the conclusion is concerned; we would like to submit that as issues have been discussed in detail in the ‘**Discussion**’, therefore, conclusion was made terse so as to avoid repetition.  The paper has been proofread for spelling and other mistakes and a number of such mistakes have been rectified. |
